# Supplementary figures and images for: Network reconstruction and systems analysis of plant cell wall deconstruction by Neurospora crassa
Source: Biotechnol Biofuels. 2017 Sep 21;10:225. doi: 10.1186/s13068-017-0901-2 (PMC5609067; doi:10.1186/s13068-017-0901-2)

**A**

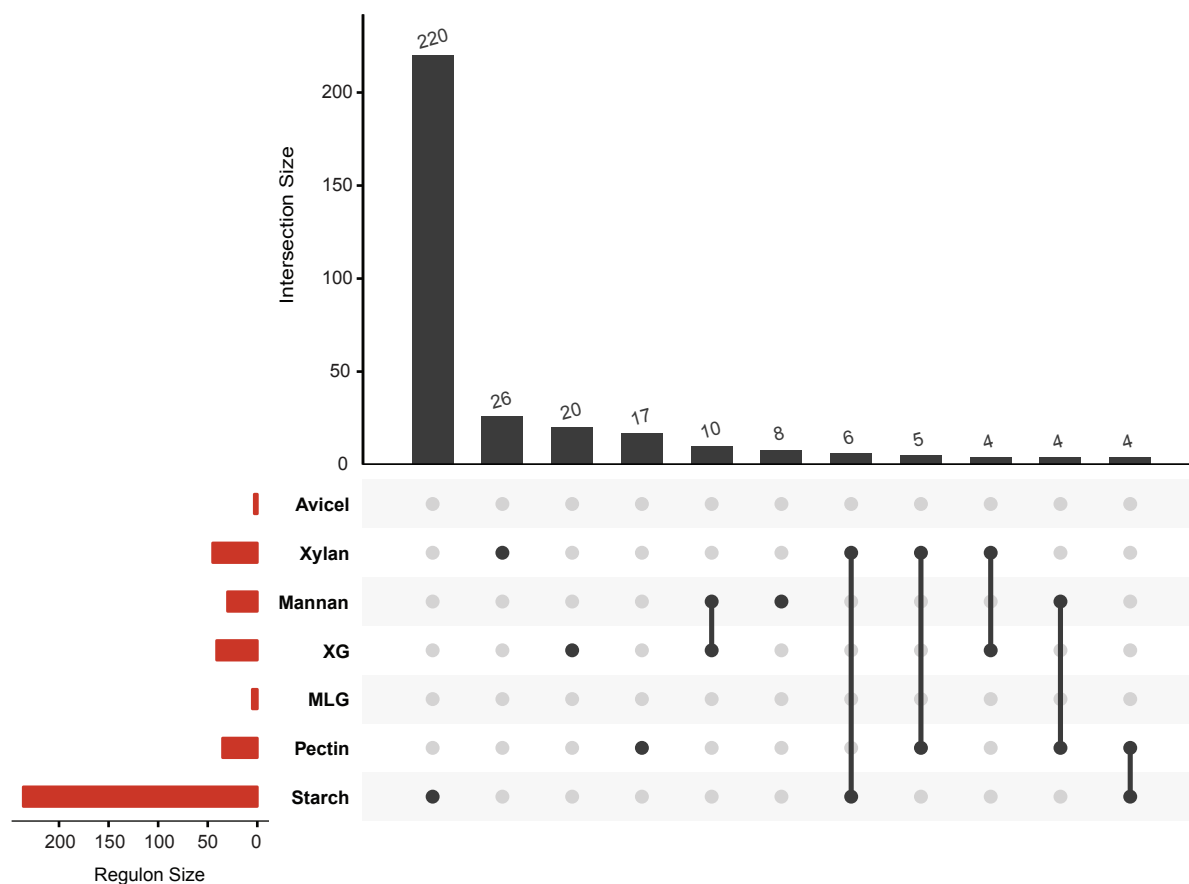

**B**

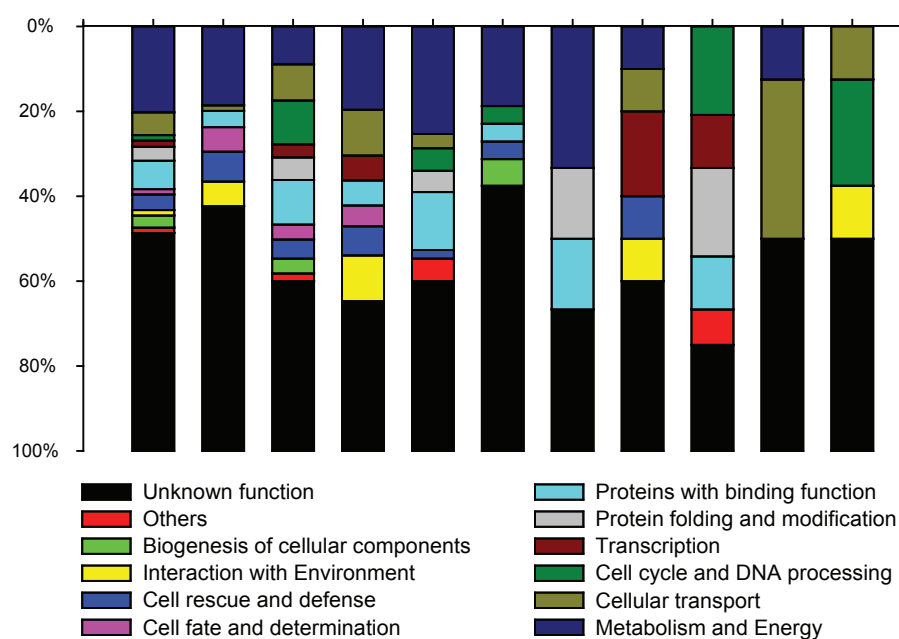

Supplement: Supplementary file 8 — Additional file 8. Comparative analysis of the down-regulons for the seven conditions. Analogous to up-regulon (for brevity referred to as ‘regulon’ in main text), the down-regulon for a growth condition can be defined as genes that were downregulated and differentially expressed in comparison to both NoC and sucrose controls. a Horizontal bar plot shows the size of the down-regulons for seven conditions. The down-regulons for Avicel, xylan, XG, mannan, MLG, pectin and starch, were determined to contain 3, 45, 41, 30, 5, 35 and 236 genes, respectively (Additional file 4). Vertical bar plot shows the 11 intersection sets among the seven down-regulons with 4 or more genes and was generated using UpSetR [108]. It is seen that 220 out of 236 genes in the starch down-regulon, 26 out of 45 genes in the xylan down-regulon, 20 out of 41 genes in the XG down-regulon, 17 out of 35 genes in the pectin down-regulon, and 8 out of 30 genes in the mannan down-regulon, have no overlap with other down-regulons. It is also seen that 10 genes are common between XG and mannan down-regulons, 6 genes between xylan and starch down-regulons, 5 genes between xylan and pectin down-regulons, 4 genes between xylan and XG down-regulons, 4 genes between mannan and pectin down-regulons, and 4 genes between pectin and starch down-regulons. b Functional category analysis [73] of the 11 intersection sets among the down-regulons for seven conditions with four or more genes. Information on the functional category of N. crassa genes was obtained from Munich Information Center for Protein Sequence (MIPS) database [73, 109]. The ‘Others’ category includes genes with functional categorization different from the 10 categories listed in the legend. The ‘Unknown function’ category includes genes with unclassified or unknown function. The relative contribution of a functional category to each set of genes is depicted with the total number of genes in each pool equal to 100%. [file 13068_2017_901_MOESM8_ESM.pdf]

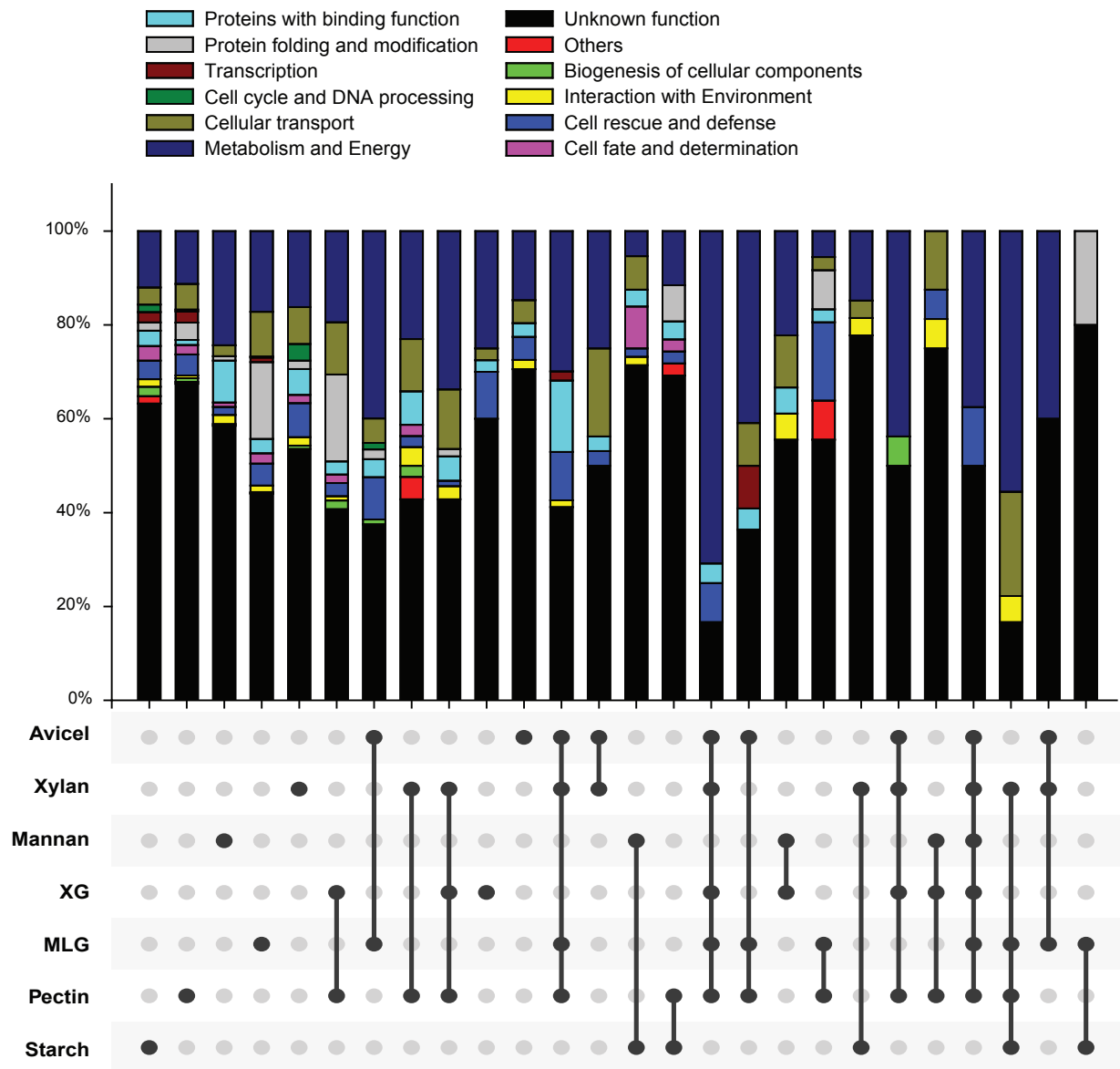

Supplement: Supplementary file 9 — Additional file 9. Functional category analysis of the 26 intersection sets among the up-regulons for seven conditions with 5 or more genes (shown in Fig. 4c). Information on the functional category of N. crassa genes was obtained from Munich Information Center for Protein Sequence (MIPS) database [73, 109]. The ‘Others’ category includes genes with functional categorization different from the 10 categories listed in the legend. The ‘Unknown function’ category includes genes with unclassified or unknown function. The relative contribution of a functional category to each set of genes is depicted with the total number of genes in each pool equal to 100%. [file 13068_2017_901_MOESM9_ESM.pdf]

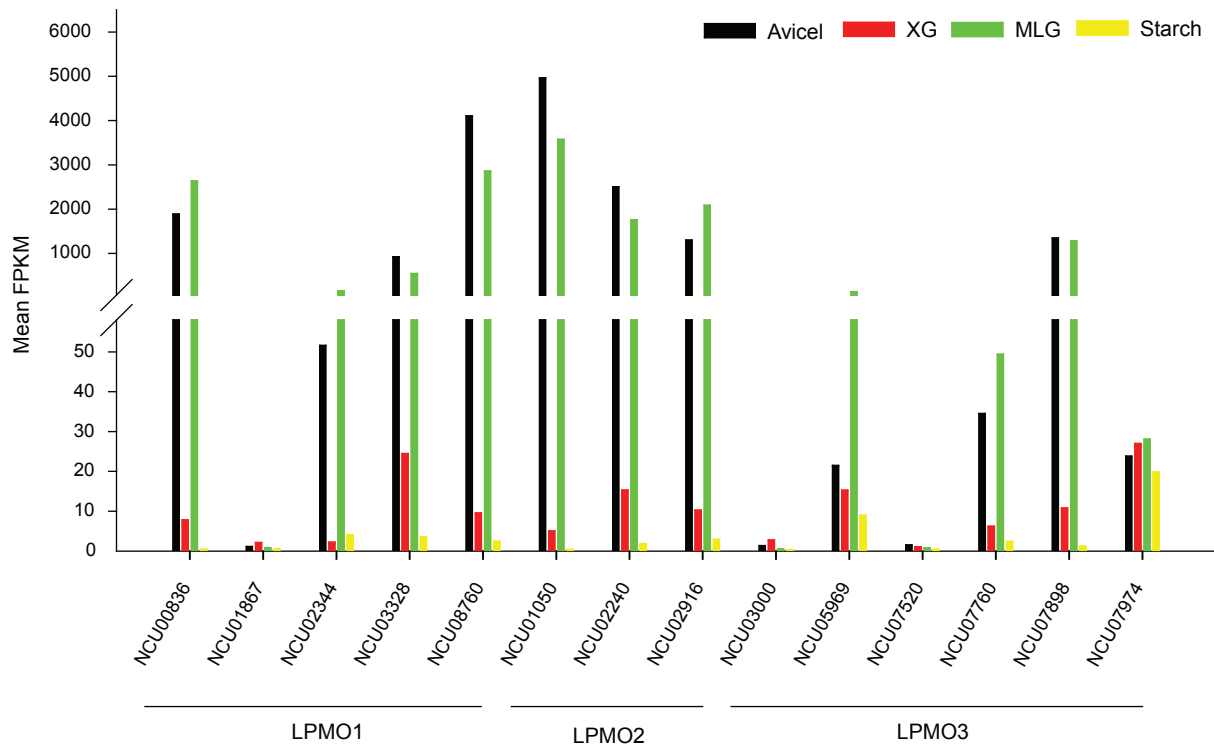

Additional File 11

Supplement: Supplementary file 11 — Additional file 11. Expression of characterized and predicted AA9 LPMOs in N. crassa grown on four plant cell wall polysaccharides with d-glucose backbone: Avicel, xyloglucan (XG), mixed-linkage glucan (MLG) and starch. In comparison to Avicel and MLG, the expression of LPMOs was much lower on XG and negligible on starch. [file 13068_2017_901_MOESM11_ESM.pdf]
